# The illusion of specific capture: surface and solution studies of suboptimal oligonucleotide hybridization

**DOI:** 10.1186/1756-0500-6-72

**Published:** 2013-02-27

**Authors:** Jaishree Garhyan, Raad Z Gharaibeh, Stephen McGee, Cynthia J Gibas

**Affiliations:** 1 Department of Bioinformatics and Genomics, The University of North Carolina at Charlotte, Charlotte, NC, USA; 2 Bioinformatics Services Division, University of North Carolina at Charlotte, North Carolina Research Campus, Kannapolis, NC, USA; 3 Greenwood Genetic Center, Greenwood, SC, USA

## Abstract

**Background:**

Hybridization based assays and capture systems depend on the specificity of hybridization between a probe and its intended target. A common guideline in the construction of DNA microarrays, for instance, is that avoiding complementary stretches of more than 15 nucleic acids in a 50 or 60-mer probe will eliminate sequence specific cross-hybridization reactions. Here we present a study of the behavior of partially matched oligonucleotide pairs with complementary stretches starting well below this threshold complementarity length – *in silico*, in solution, and at the microarray surface. The modeled behavior of pairs of oligonucleotide probes and their targets suggests that even a complementary stretch of sequence 12 nt in length would give rise to specific cross-hybridization. We designed a set of binding partners to a 50-mer oligonucleotide containing complementary stretches from 6 nt to 21 nt in length.

**Results:**

Solution melting experiments demonstrate that stable partial duplexes can form when only 12 bp of complementary sequence are present; surface hybridization experiments confirm that a signal close in magnitude to full-strength signal can be obtained from hybridization of a 12 bp duplex within a 50mer oligonucleotide.

**Conclusions:**

Microarray and other molecular capture strategies that rely on a 15 nt lower complementarity bound for eliminating specific cross-hybridization may not be sufficiently conservative.

## Background

DNA microarrays remain a popular technology for measuring gene expression and other global properties of the genome, with over 2200 experiments representing tens of thousands of samples published in ArrayExpress [1,2] so far in 2012. Even as next-gen sequencing technology has begun to supersede microarrays for such measurements, many researchers still rely on them for various applications. For instance, in a recent, highly cited sequencing study of plague (Y. pestis), Bos et al. [3] used microarrays as a capture technology to concentrate samples for sequencing. Stransky et al. [4] recently used microarrays for the purpose of screening head and neck tumor samples prior to sequencing; these are just two of many examples. In this paper, we report on the potential for stable duplex formation between partially complementary oligonucleotides and unintended DNA targets, which has significant implications for their ability to capture non-target material whether in the context of a wholly microarray-based experiment, or a sample concentration protocol.

The conventional wisdom surrounding design of oligonucleotide microarrays, specifically those of the type that rely on 50–60mer oligonucleotides for detection, was established in the early 2000s. Cross-hybridization is defined as a specific side reaction between a probe and an unintended target to form a stable duplex, and microarray design pipelines generally attempt to avoid this either by screening for defined levels of sequence complementarity, or by using a thermodynamic cutoff, though in the latter cases, sequence complementarity is often used as a pre-screen.

A common criterion for microarray design, used in many oligonucleotide design software pipelines [5] either as a pre-screen or as the sole predictor of potential cross hybridization, is based largely on an early paper from Kane et al. [6] and is generally referred to as Kane’s first criterion. This criterion, that eliminating stretches of apparent complementarity longer than 15 nucleotides between a probe and unintended targets will eliminate cross-hybridization, is very convenient for microarray designers, because it justifies the use of fast suffix-tree based methods for sequence screening using a word size that will automatically exclude most entirely random short alignments. While shorter complementary stretches can be identified using Smith-Waterman alignment or other approaches [7,8], it may be impossible to eliminate shorter cross-hybridizing stretches for every gene in expression experiments, due to the relatively limited sequence space explored by mRNAs and noncoding RNAs [8].

Kane’s criteria have always been somewhat problematic, because in the original experiment, the effects of complementarity may have been confounded with unimolecular structure formation in the reagents. Later studies that attempted to validate the Kane criteria did not challenge the established lower bound of complementarity using constructed complementary stretches shorter than 15 nt. [9] The behavior of competing closest thermodynamic near neighbors has been investigated by Chou et al. in [10], but the focus there was competition between the intended target and the thermodynamically nearest neighbor, rather than on the general hybridization potential of partially matched duplexes. The results presented here suggest that Kane’s first criterion may be insufficiently conservative to eliminate significant specific cross-hybridization in surface hybridization experiments.

To explore the hybridization potential of suboptimal duplexes, we first performed computational modeling of duplex formation in partially complementary oligonucleotide pairs, using the DNA Software hybridization modeling package based on the work of SantaLucia et al. [11,12] The interactions of 50mer oligonucleotides containing complementary stretches of nucleotides from 6 nt to 25 nt at different positions in the sequence were modeled, alone and in the presence of a perfect match competitor at different relative concentrations. The predicted behavior of these oligonucleotide pairs indicated that we could expect significant signal from specific, partial cross-hybridization, due to complementary stretches forming as few as twelve consecutive base pairs, and detectable hybridization even due to a complementary 9mer in an otherwise anticomplementary probe-target pair.

We then selected a typical perfect match oligonucleotide duplex pair, with average GC content and T_m_ relative to a probeset designed for the *E*. *coli* genome. We created permutations of the sequence of one of the perfect match partners, leaving a continuous complementary stretch of varying length either in the center of the molecules, or positioned near one end. We synthesized the perfect match partners and a selection of the permuted, partially matched sequences to observe the hybridization behavior of these sequences in solution and on the array.

Here we report the results of hybridization of those permuted oligos to their binding partner, both in solution, and at the microarray surface. The analysis of this permuted oligo pair confirms that a complementary stretch of nucleotides as short as 12 bp may result in the appearance of significant signal from an unintended binding partner, especially in the absence of the intended target. This illusion of specific capture has the potential to give rise to incorrect interpretations of expression data, but it is potentially a problem even in sample concentration applications, where a transcript with relatively little complementarity may be captured and interpreted as if it were part of the intended target, when in fact it is not.

## Results and discussion

### A genome-wide modeling survey of suboptimal hybridization

To generate a representative probe-target pair for this experiment, we designed a set of probes for the *E*. *coli* genome using commonly-used array design applications to screen sequences for uniqueness and thermodynamic uniformity, as described in the Methods. We analyzed the distributions of melting temperature (T_m_) values and sequence composition within this set, and chose probes that fell in the center of these distributions, then we examined candidate pairs to eliminate those with specific substrings that were likely to give rise to thermodynamic nonuniformity (e.g. GGGG). [13] The probe chosen (1) and its complementary target subsequence, were synthesized, along with several permutations of one sequence, to produce partially complementary pairs.

(1) CGATCTGGTACTGAGTTACACCACCTCTCCG GCTTATCACATTCTCGAAG.

### Modeled behavior of representative oligonucleotide pairs

**Figure 1:**
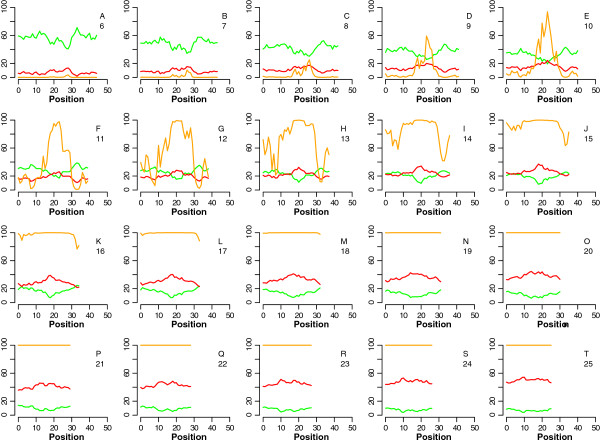
**Results of OMP simulation of hybridization between a probe and a partially matched target.** The X axis value is the starting position of the complementary stretch in the sequence; the Y axis is fraction bound (F_B_). In all simulations, the probe is present in excess of the target; the perfectly matched target is present in 2-fold excess of the partially matched target. The orange line in each plot shows the fraction of partially matched target bound to the probe (F_B_), while the red and green lines show the absolute ΔG and absolute T_m_ difference between the mismatch and perfect match pairs, respectively. Parts A-T show the change in binding of the mismatch as the complementary stretch length increases from 6 to 25. The complementary stretch length is shown under each panel’s label.

To assess the hybridization potential of partial duplexes, we modified one member of the sequence pair, leaving a single continuous stretch of sequence complementary to the binding partner. We varied the length of the complementary stretch and the starting position of the complementary stretch within the sequence. Hybridization between the designed probe and the permuted target sequence was simulated using the Oligonucleotide Modeling Platform (OMP) from DNA Software, as described in the Methods section. Figure 1 shows hybridization profiles for partially complementary pairs, for complementary stretches between 6 and 25 nt in length, and with the complementary stretch at all possible positions within the sequence. In Figure 1, the concentration of the perfect match target is in 2-fold excess of the partial match target, with the probe present in excess. Additional file 1 Figure S1 shows similar hybridization profiles for partial match target equal in concentration to the perfect match. The modeling results suggest that, depending on the relative concentration of a suboptimally-matched target and the intended perfect match, as well as on the position of a complementary stretch within the sequence, an unintended binding partner could give rise to signal at a 50-mer probe with as few as 9 consecutive matches present.

### Solution behavior of representative oligonucleotide pairs

To investigate how those permutations will affect duplex formation in solution, we chose a subset of the permuted targets that were simulated in the previous section along with the perfect match target and its probe. Partial matches to the original probe (1) with complementary stretches of 9, 12, 15, 18 and 21 nucleotides were synthesized. In addition, we intentionally picked pairs where the complementary stretch was located either near the center of the oligo pair (central), or near one end of the pair (terminal). We then measured duplex formation in solution and determined the melting temperature of each of these suboptimal duplexes.

**Figure 2:**
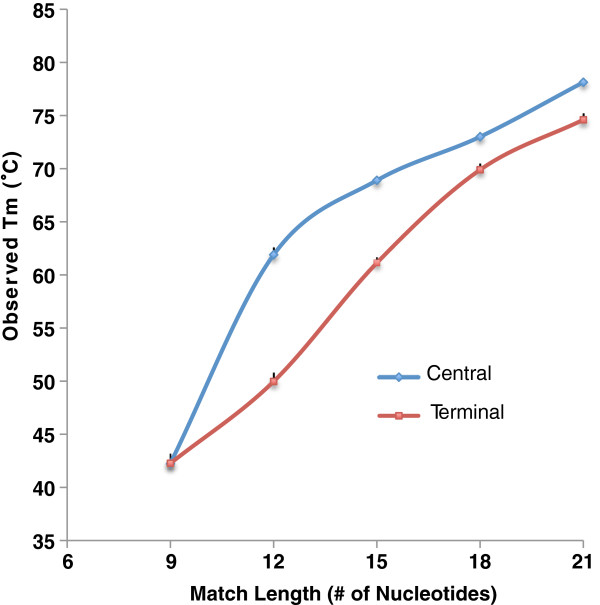
**Observed solution T**_**m **_**of partially matched probe-target duplexes with varying complementary stretch.** Complementary stretches were positioned at central (indicated by blue) or terminal ends (indicated by red). Stretch length varied from 9 to 21nt. Each target was mixed with the probe to form a duplex and then allowed to undergo melting followed by denaturation to obtain melting temperature. Error bars are standard deviation among replicates for probe-target duplex.

**Figure 3:**
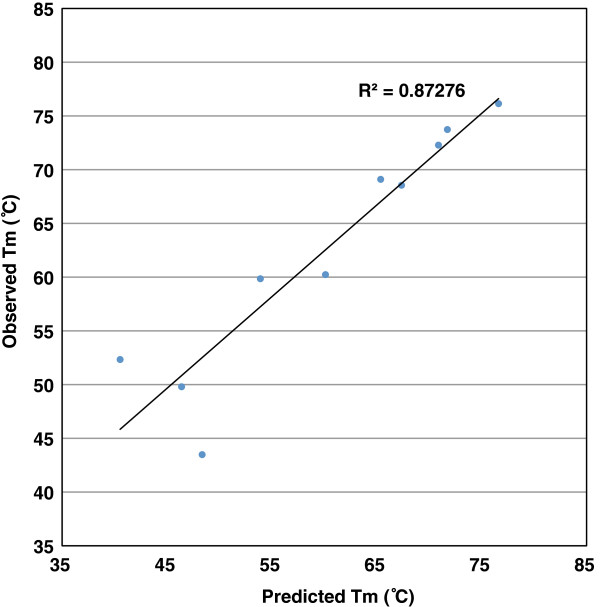
**Predicted vs. observed melting temperature (T**_**m**_**) of central and terminal probe-target duplex in solution state.**

Figure 2 shows the observed melting temperature in solution for 10 pairs of suboptimally matched oligonucleotides having stretches of complementarity ranging in size from 9 nucleotides to 21 nucleotides. T_m_ is measured for central and terminal match positions. The original perfect match duplex had observed T_m_ of 87.95°C, modeled T_m_ of 69.76°C. Across the entire set of experiments, observed T_m_ is in better agreement with modeled T_m_, with R^2^ = 0.87 (Figure 3).

As expected, the measured solution melting temperature of long oligos forming short duplexes is low relative to the perfect match T_m_. The solution melting temperatures of oligo pairs containing terminal duplexes of 9 or 12 nucleotides, and of the pair containing a central duplex of 9 nucleotides, are below 55°C. When we consider the potential significance of such pairings to the microarray context, it is clear that such pairings are likely to have minimal impact on the signal in protocols where the hybridization temperature is 55°C or above. Hybridization temperatures ranging from 50–60°C are commonly used in protocols for processing long oligo (50–60mer) microarrays. The solution melting temperature of pairs containing longer central complementary stretches rises to near 70°C when a central complementary stretch of 15 nt or a terminal stretch of 18 nt is present. The impact of these partial matches on the experimental outcome will be increased or lessened depending on the conditions, but the potential for significant unintended hybridization in solution is present, even with complementary stretches that would not be ruled out by Kane’s commonly used criteria.

### Microarray surface behavior of representative oligonucleotide pairs

We carried out multiple sets of experiments in order to test the behavior of the same set of 50mer probe-target pairs used in the simulation and the solution experiments, on the microarray surface. The same probe-target pairs used in the solution state experiments were used, with the original probe attached to the microarray surface and the series of permutated targets applied in the hybridization reaction, either alone, or in the presence of an equimolar concentration of the perfect match target.

**Figure 4:**
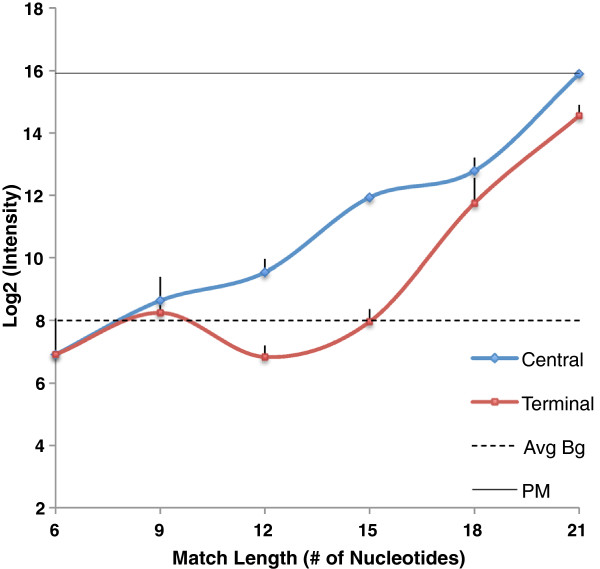
**Surface-attached probe response to 50mer targets with complementary stretch from 6 to 21 nt.** Here we show a comparison between probe response to targets with centrally located matches (central shown in blue) with targets with matches located in 3’ end or 5’ end (red). The dotted line represents average background across all the targets used and the solid line represents probe response to its 50mer perfect match. Error bars indicate the standard deviation among replicated arrays.

In the first set of experiments we chose a standard hybridization temperature of 60°C and hybridized individual targets to the probe. Figure 4 shows that the target with a central complementary stretch of 12 nt match length begins to show signal intensity significantly above the background level, while targets with complementary stretches shorter than 12 nt fall below the background level. A significant increase in the intensity is observed as the match length increases from 12 to 21, at which point the signal from the partial match in isolation approaches the signal from the perfect match (PM) target. As expected, signal intensities for targets with a terminal complementary stretch fall below signal intensities for their counterparts with a central complementary stretch of the same length. A terminal complementary stretch of 12 nt gives signal below the background level. This is congruent with prior experiments, in which the position of match plays an important role in determining the signal intensity [14,15].

**Figure 5:**
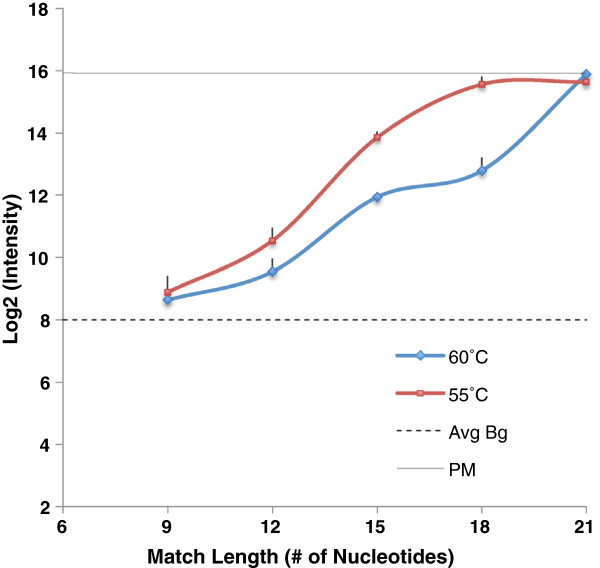
**Probe response to targets at two different hybridization temperatures of 55°C (represented in red) and 60°C (represented in Blue).** Targets with complementary stretch of 9 to 21 nt were hybridized with surface attached probe at hybridization temperatures of 55°C and 60°C for 18 hrs followed by standard washing and drying procedure. Average background is indicated by dotted line. Probe response to perfect match at 55°C and 60°C are not significantly different (Data not shown). Error bars represent standard deviation across the replicate arrays.

We then repeated the above experiment at 55°C with the targets having a central complementary stretch. Figure 5 shows that at hybridization temperatures of 60°C and 55°C, targets with a central match of 12 nt or more give significant signal intensity above the background level. The increase in intensity observed as the central match stretch increases from 12 to 21 nt is consistent in the 55°C experiments.

**Figure 6:**
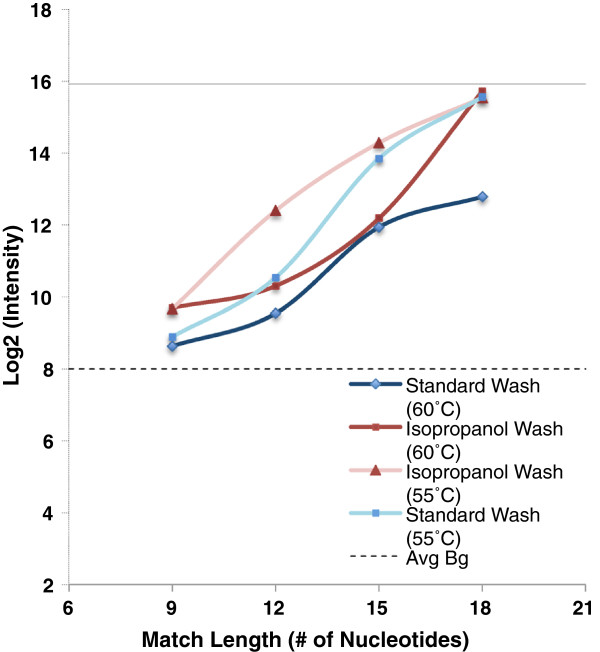
**Effect of isopropanol wash on measured probe response to target.** Targets with were individually hybridized with surface-attached probe using our standard 18-hour protocol, followed by either isopropanol wash (red) or standard wash (blue). Signal response at both 55°C and 60°C are reported. Average background is indicated by the dotted line and perfect match response is shown by the solid line. Error bars represent the standard deviation across the arrays for each probe target duplex set.

In some experimental protocols, isopropanol washes are now used to reduce the loss of intended target material from the microarray slide during washing. We repeated measurements at both temperatures, using an isopropanol washing procedure. The expected result was observed at both 55°C and 60°C; isopropanol washing resulted in significantly increased signal from suboptimal matches with complementary stretches in the 12 nt-21 nt range, suggesting that signal due to formation of partial duplexes with unintended targets contributes to the signal differences observed between isopropanol-washed and buffer-washed arrays (Figure 6).

**Figure 7:**
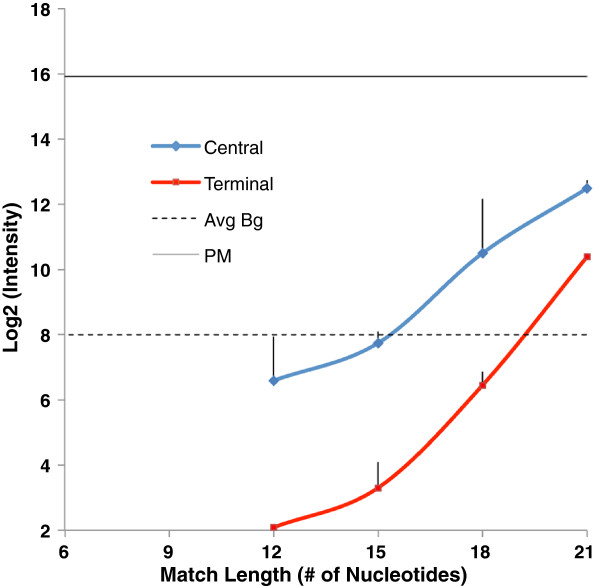
**Probe response to central and terminal targets in the presence of equimolar concentration of unlabeled perfect match.** Average background is represented by dotted lines. Error bars indicate the standard deviation of replicated arrays.

In our simulations, the effects of suboptimal binding are predicted to be mitigated, though not completely eliminated, by the presence of the perfect match target in equimolar concentration. On the array, we performed competition experiments to determine the signal due to binding a partially matched target, in the presence of an equimolar concentration of the intended target. The partially-matched target is labeled, while the perfect-match competitor is unlabeled. Figure 7 shows that the perfect match target, if present in sufficient quantity, will outcompete the partially-matched target, if the stretch of complementarity between probe and partially-matched target is less than 18 nt. If a stretch of complementarity 18 nt or greater is present, the signal due to the partially-matched target climbs above background, even in the presence of an equal quantity of perfectly match target, and thus such matches can contribute to error in the signal.

## Conclusions

We conclude that targets with very low levels of complementarity are capable of producing signals indistinguishable from a low intensity perfect match signal under common conditions of microarray experiments using longer (50–60 nt) oligonucleotide probes. The commonly used cutoff of 15 consecutive complementary bases may not be sufficient to eliminate unintended hybridizations, especially if the complementary stretch occurs in the center of the probe-target pair. These effects are mitigated but not eliminated by presence of a competing quantity of the perfect match target.

In practical terms, this suggests that it will be very difficult to eliminate cross-hybridization in microarray design. Not only should we consider the potential formation of 12- to 15-mer partial duplexes, but other, more complex interactions should be considered as well. The second of Kane’s two criteria is that a target with more than 75% total complementarity to another probe should be considered a risk for cross-hybridization, but we have observed that it is possible to design probe-target pairs that pass both these tests yet still yield signal under standard hybridization conditions. More extensive experiments would be required to parameterize a general model of competition at the low end of complementarity, but the results reported herein suggest that further investigation of the issue and construction of such models may be a worthwhile endeavor.

### Implications for microarray applications

Modeling of suboptimal hybridization suggests that hybridization-based assays using 50-mer oligonucleotides may sensitively and specifically capture not only perfect match binding partners, but significant quantities of specific, partially complementary sequence as well. Assays on a representative, GC balanced 50-mer oligo, its perfect match binding partner, and a variety of suboptimal matches demonstrate that significant signal may arise from formation of partial duplexes, even in competition with the perfect match duplex. Signal from a partial duplex may also appear indistinguishable from low signal from a perfect match, when the perfect match is absent.

In expression microarray experiments, it is well known that hybridization based expression values do not consistently agree with “gold standard” measurements from quantitative PCR, and it is likely that unexpected side reactions are a component of these differences. But the implications of this study are broader. Since the advent of second generation sequencing, RNA-Seq has increasingly replaced microarrays as the method for expression profiling. However, arrays are still used in genomics and transcriptomics as a preliminary filtering step prior to the sequencing assay. In these contexts, the capture of partially complementary material at low concentrations may confound the results of an assay.

## Methods

### Hybridization simulations

Given the differences that exist between solution hybridization (melting experiment) and on chip hybridization, two sets of hybridization simulation were done. The first set was designed to mimic solution hybridization where all strands have the same concentration. The second set was designed to mimic on chip hybridization where one strand (probe) is present in greater concentration than the other strand (target).

For the first set, multiple hybridization runs were done at different concentrations (remember: both strands have the same concentration) starting from 3 × 10^-11^ up to 1 × 10^-6^ M for each strand. The sets were hybridized first as 1:1, where only one 5`-3` strand and one 3`-5` strand were used (no competition) as illustrated in Figure 1A. The same sets were hybridized again in the presence of competing strands, as illustrated in Figure 1B.

All simulations were done using the Oligonucleotide Modeling Platform Developer Edition for Linux (OMP DE) release of 25-Feb-2010.

### Solution experiments

#### Sample preparation

HPLC purified single stranded unlabeled oligonucleotides were manufactured by Operon Biotechnologies (Huntsville, AL). Stock oligos were suspended in 1 M Sørensen’s sodium phosphate buffer solution (72 ml of 2 M Na_2_HPO_4_ (Sigma Aldrich) and 28 ml of NaH_2_PO_4_ in 100 ml ddH_2_O, degassed by freeze-thaw method) pH 7.2. [16] 300 ul of target-probe mix each at a concentration of 2 uM diluted in degassed phosphate buffer was hand pipetted into uv-silica cuvette (path length = 1 cm, Beckman Coulter Inc., part # 523878) and sealed immediately with a stopper to avoid evaporation.

#### Melting temperature in solution state/solution phase hybridization

UV melting curves were obtained using a DU-800 spectrophotometer (Beckman Coulter Inc., Fullerton, CA, USA) connected to a Peltier High Performance Temperature Controller used for controlling sample temperature, following the protocol of Owczarzy et al. in [17]. Each cuvette containing a probe-target mixture was placed in temperature controlled Peltier six-cell holder. Sørensen’s sodium phosphate buffer solution was used as the blank. Samples were heated at the rate of 0.8°C/min over the temperature range from 15 to 90°C and absorbance was measured every minute at 260 nm to obtain cooling / denaturation curves. The specifications used during the runs were: Start temp: 15°C, Start dwell: 0 min, End temp: 95°C, End dwell: 5 min, Ramp rate: 0.8°C/min and Reading rate: 0.8°C/min. Samples were then cooled down in a reverse reaction and absorbance was recorded every minute following the same specifications. The three replicates of each probe-target pair were carried out in different cell-positions in the Peltier holder. Subsequently, heating and cooling absorbance vs. temperature curves and derivative curves were collected for each probe-target pair. The melting temperature reported is the maximum of the first derivative of the melting curve; errors reported are standard error based on three replicate measurements. The melting curve analysis was implemented in Microsoft Excel.

### Array experiments

#### Oligo selection

The sequences selected in this experiment were derived from the *E. coli* K12 strain. A commonly used microarray design application, YODA [18], was first used to generate probes screened for uniqueness and self-complementarity following Kane’s criteria. A subsequent round of screening using another program, PICKY [19], removed probes that were not thermodynamically uniform. We chose one probe-target pair that was average in composition and melting temperature relative to the entire design. The sequence designated as the “probe” remained constant throughout the experiment, while the 50-mer complementary sequence designated as the “target” was subject to permutation.

#### Generation of target mixtures

Target oligonucleotides modified with cy3 attached at the 5’ end were manufactured by Operon Biotechnologies (Huntsville, AL). They were re-suspended to 100uM concentration in 3× SSC. Targets were diluted to a concentration of 500 pM using a hybridization solution (0.5 mg/ml salmon sperm, 6 × SSC, 0.05% SDS) to prepare a final target hybridization solution. The final target hybridization solution was subsequently heated to 95°C for 5 minutes, and then chilled on ice for 5 minutes before addition for hybridization.

#### Design of microarray slides

A 4 × 4 array was printed on each slide. Each array had four replicate spots of each of four samples -- buffer 3 × SSC, a negative control oligo, a labeled sentinel oligo, and the probe specific for the target mixtures used in these experiments. The negative control and sentinel probes were designed based on sequences of *A*. *thaliana*. Stringent selection criteria were used to rule out formation of subduplexes with the target sequences used in the experiment, followed by simulation in OMP to rule out unforeseen nonconsecutive interactions with the target sequences.

#### Fabrication of microarray slides

Probes with amino-C6 linkers at the 5’ end were manufactured by Operon Biotechnologies (Huntsville, AL). Probes were re-suspended to 100uM concentration in 3 × SSC and brought to a final concentration of 20uM with 3 × SSC. 10 ul of mel_ex (minimum nucleation) probe, positive control, negative control probe and print buffer controls were transferred to a 384-well plate (Whatman Inc., NJ). These composite plates were then used as source plates for printing an array of 20 spots (4 × 5) onto epoxide coated slides (Corning cat# 40041) using quill stealth microspotting SMP3B pins (Arrayit Corp) mounted in a BioOdyssey Calligrapher miniarrayer from Bio-Rad (München, Germany). This printing regime yields spots of approximately 110 μm in diameter. The printing conditions were set via the Bio-Rad Calligrapher Software. To avoid carryover, pins were treated for two cycles with a 5 s wash and a 5 s dry between print loads. The air temperature inside the spotting chamber was 20°C and the humidity was set to 55%. For achieving covalent attachment of oligonucleotides, printed arrays were incubated in the Calligrapher for 1 hour and then transferred to a humid chamber at 42°C for 18 hrs. Subsequently, slides were stored in a dark desiccator at a temperature of 4°C until required.

#### QC of printed slides

For evaluation of in-house printed arrays, three QC assays were performed: SYBR Green II staining, a 9mer accessibility assay, and a TDT assay which demonstrates accessibility of the free end of the probe. One slide from each batch was stained with SYBR Green II to ensure spot uniformity. Arrays were stained with 1:10000 dilution of SYBR Green II (Invitrogen cat# S7564) for 2 minutes and subsequently washed 3 times with 1XTBE buffer, dried and scanned. Next, probe accessibility was tested with a 9mer assay in which arrays were incubated with 9mer solution (FMB Microarray QC kit cat #MQC) for 1 minute followed by three rinses with 1 × SSC. [20] Subsequently they were dried and scanned. The third QC assay was a TDT end labeling assay, in which one slide from each batch was covered with 1 uM cy3 dCTP (GE cat# PA 53021), 1× TDT reaction buffer and 1 unit dTDT (Affymetrix PN# 72033) [21] and incubated at 37°C for 25 min. Slides were washed twice with 0.1 × SSC and dried for scanning. After each batch of arrays passed the three QC assays they were used for hybridization experiments.

#### Array hybridization

The slides were placed in a HS 4800 Pro Hybridization Station (Tecan, Mannedorf, Switzerland), which had been preheated to 55°C. All wash solutions were also preheated by the hybridization station. The slides were then wetted by a brief rinse with a Hybridization Wash solution (0.5 × SSC, 0.005% SDS), and then blocked with BlockIt solution (Cat #BKT, ArrayIt, Sunnyvale, CA) for 30 minutes. The slides were then washed again for 2 minutes with the Hybridization Wash solution. 60 μL of target solution (concentration of 500 pM) was then added and the slides were hybridized for 18 hours at 60°C or 55°C with agitation set at medium intensity. After the hybridization step, the slides were washed three times in the Hybridization Wash solution for 30 seconds and cooled to 55°C, washed for 2 minutes with the Hybridization Wash solution and cooled to 50°C, washed with TE for 30 seconds and cooled to 45°C, washed with 0.5× TE for 30 seconds and cooled to 40°C, washed with 5% alcohol (from 200 proof molecular grade Ethanol, Sigma Aldrich Cat# E7023-500 mL, St. Louis, MO) for 1 minute and cooled to 30°C, and finally washed twice with ddH_2_O for 40 seconds and cooled to 25°C. After these washes, the slides were dried under ultra-pure nitrogen for 3 minutes as previously described by Gharaibeh et al. in [22]. For isopropanol washes after 18 hours of hybridization, slides were manually dipped in isopropanol (Sigma Aldrich Cat #I9516, St. Louis, MO) for 2 seconds and dried with our standard procedure.

#### Image acquisition and data analysis

Slides were scanned with the 532 nm laser, using a 575 nm filter, at 5 μm resolution under autofocus mode in the LS Reloaded Scanner (Tecan, Mannedorf, Switzerland). Scanned images were saved as TIFF files and then signal intensities were quantified using SPOT (CSIRO, Sydney, Australia). The quality of each array and its spots were determined following procedures described by Gharaibeh et al. in [16]. Data was analyzed using Microsoft Excel. Curves are constructed using the smooth marked scatter function and thus do not represent a model fit to the data; error bars represent standard error computed on three replicate measurements. SPSS (version 17.1) was used to perform t-tests and analysis of variance.

## Competing interests

The authors have no financial or other conflicts of interest in the publication of this manuscript.

## Authors’ contributions

JG carried out the fabrication of microarrays used in the study, performed the hybridization experiments, analyzed the data, and helped to draft the manuscript. RG designed the reagents and performed in silico simulations of hybridization. SM carried out and analyzed the solution hybridization experiments. CG conceived of the study, and participated in its design and coordination and helped to draft the manuscript. All authors read and approved the final manuscript.

## Supplementary Material

Additional file 1: Figure S1 Results of OMP simulation of hybridization between a probe and a partially matched target. The X axis value is the starting position of the complementary stretch in the sequence; the Y axis is fraction bound (F_B_). In all simulations, the probe is present in excess of the target; the perfectly matched target is present in equal concentration as the partially matched target. The orange line in each plot shows the fraction of partially matched target bound to the probe (F_B_), while the red and green lines show the absolute ΔG and absolute T_m_ difference between the mismatch and perfect match pairs, respectively. Parts A-T show the change in binding of the mismatch as the complementary stretch length increases from 6 to 25. The complementary stretch length is shown under each panel’s label. Click here for file
